# Practical Pathology of the Mucous Membrane of the Nose, Throat, and Ears of Infants

**Published:** 1896-05

**Authors:** Thomas F. Rumbold

**Affiliations:** St. Louis, Mo.


ATLANTA
Medical and Surgical Journal.

VOL. XIII. MAY, 1896. No. 3.

LUTHER B. GRANDY, M.D., MANAGING EDITOR.

M. B. HUTCHINS, M.D., BUSINESS MANAGER.

COLLABORATORS:
H. V. M. MILLER, M.D., LL.D., A. W. CALHOUN, M.D., LL.D., VIRGIL O. HARDON, M.D., FLOYD W. McRAE, M.D., AND DUNBAR ROY, M.D.

### ORIGINAL COMMUNICATIONS.

### PRACTICAL PATHOLOGY OF THE MUCOUS MEMBRANE OF THE NOSE, THROAT, AND EARS OF INFANTS.

BY THOMAS F. RUMBOLD, M.D.,
ST. LOUIS, MO.

While the subject under consideration is the pathological condition of the mucous membrane of the nose, throat, and ears, yet as it is well known that when the inflammation of these organs becomes severe or is protracted, it implicates other organs—the eyes, the brain, the lungs, the stomach, or portions of the nervous system, consequently these secondarily affected parts must necessarily be alluded to in connection with the primary subject.

The value of the study of the pathological conditions of the mucous membrane of the nose, throat, and ears is conceded by all who have had even a short experience in the ailments of these organs. I fully believe that most of the advancement that is to be made in practice is to come through successful investigation in the line of pathology. This study is not to be confined to the consideration of the morbid phenomena merely, but as far as possible to be extended to the *essential* nature of the morbid actions themselves. It is along this line that investigation should be pushed. The facts thus gathered should be *utilized* as indications of the proper course and method of treatment. Of course the normal action of the part affected must always be borne in mind while studying its diseased action, for it is only by comparing the action of the part in its healthy and diseased states that a conclusion can be arrived at as to the degree of departure from health.

*The Inflamed Mucous Membrane of Infants* to the third year of age will be the subject considered in this article. The mucous membrane of the nasal passages is the tissue upon which the inflammation first shows itself—up to this time the infant has been *healthy in every respect*—consequently the actual condition of this tissue will first be described.

The manifestations of disease consist in an increased flow of apparently normal mucus, and a slight increase of the color of the mucous membrane, soon followed by its becoming more or less thickened. From this stage the morbid processes are characterized by conditions that are apparently very diverse. Sometimes the papillæ of the mucous membrane of the anterior portion of the inferior turbinates are temporarily enlarged, producing an irregularity of surface. The irregularity is due to swollen submucous glands, and positively indicates a subsidence of a severe inflammation that has implicated the whole of the passages.

Sometimes, but rarely, there is, even at this age, an atrophy of the mucous membrane, but this condition is always preceded by comparatively long-continued as well as excessive engorgement of the blood-vessels, the atrophy being due to a greatly diminished blood-flow in the part, a circulation that is not sufficient to maintain even the normal nutrition of the part, much less its former swollen condition. I doubt very much that this atrophy is permanent. Even without treatment recovery has frequently been observed to take place.

Hyperemia, then, is the first objective symptom of this disease, and is occasioned by a dilation of the blood-vessels as a result of paresis of the sympathetic nerves connected with them. The mechanism of this condition is briefly as follows: While mucous membrane is the first to show objective symptoms, the sensory nerves of the integumentary surface of the body, especially that of the head, face, neck, and hands, is the first part irritated by the cold. These sensory nerves, which are afferent in their action, transfer this *irritation* inward to the cervical sympathetic ganglia, which in turn reflect the *irritation* outward through their efferent nerves to the mucous membrane. It is thus seen that the sensory nerves first receive the irritation: They do not show it, but transfer the injury received to the sympathetic nerves, which do manifest it on the mucous membrane. It follows that if one will take care to guard the sensory nerves from irritation, the sympathetic nerves will remain in a healthy condition, for these nerves cannot be diseased unless injured through the sensory nerves. Thus pathology teaches a most valuable lesson in *hygiene.* If the infant takes cold from the irritation occasioned by inspiration of a cold draught of air into its nostrils, then the sensory nerves of this surface play the same part that was performed by those of the integumentary surface, resulting in a swollen condition of the mucosa of the part. The swelling is due to the increased diameter of every blood-vessel, and this condition is, as already stated, a result of a paretic condition of the muscular coat of the blood-vessels. This phenomena in total is called inflammation.*

*Growths.* This condition is followed by an apparent retardation of the blood-flow. The effect of this retardation is still greater dilation of the blood-vessels and capillaries, so that much more blood is retained than is required. With this excess of retention of blood, enlargements or growths *always do and must take place,* simply because of the presence of this nutritive fluid, the

*I stated in my private lectures on Rhinology to physicians in the fall of 1873, that "A cold was due to a diseased condition of the sympathetic nerves." (See page 718, Treatise on Catarrhal Diseases of the Nose, Throat and Ears.—Rumbold, 1888.) I arrived at this conclusion from the study of the histological anatomy, physiology, and pathology of these organs, and from observations made in practice, almost entirely limited to the diseases affecting these organs, during the previous eleven years. Most of the facts from which I draw this inference are clearly stated in Todd and Bowman's Physiological Anatomy, 1855, but they are not placed in such relation, the one to the other, as to show the important fact that the paresis of the sympathetic nerves and consequent inflammation of mucous membrane is occasioned by limitation of the sensory nerves of the surface of the body.

blood. Excess of blood always produces excess of growths. The size of the enlargements or growths will vary according to the age of the inflammatory action, and according to the condition of the mucous membrane affected, that is, whether it has or has not previously been slightly or greatly inflamed. In the infant these changes are not very manifest, as it has not lived long enough to allow the excess of blood-supply to produce growths of great size

The most manifest change of the mucous membrane of the infant is the enlargement of the blood-vessels; this is sufficient to allow the transudation of serum. The serum transudes mainly from the capillaries and small veins, but also from the small arteries. This serum differs from blood-serum in being of lower specific gravity, because it contains more water. The greater the distention of the blood-vessels, the more nearly does this serum resemble blood-serum and the greater is the amount of albumin which it contains. If the pressure on the walls of the blood-vessels be great, as in severe acute inflammation, the serum may yield a fibrinous coagulum. When inflammation of this severity occurs in the Eustachian tubes and fibrin is thrown out, it closes the tubes, and then very serious ear trouble is sure to follow, frequently resulting in deafness or deaf-muteness or death. The latter two results are only averted by paracentesis of the membrane tympani, or by its rupture occasioned by ulceration.

*The plexus of vessels*, that is so plainly seen in the adult turbinates, on dissection, is not seen in the infant. In a few heads that I have examined, that were nearly three years old, the blood-vessels were beginning to form plexuses. It is easy to distinguish the arteries from the veins even in the third year of age, and even when the plexus of the vessels is partially formed. This condition of the blood-vessels and mucous membrane is also seen in the nasal passages of the healthy dog, sheep, hog, goat, cat, mouse, rat, rabbit, squirrel, cow, horse, etc., tending to show that the enlarged condition of the blood-vessels, that is, in the form of plexuses, is not the normal condition.

*Edema.* When the lymphatics are insufficient to remove the transuded serum by absorption, edema is the result. The passage of the serum occurs without rupture of the walls of the blood-vessels, and even blood corpuscles may pass through the coat of the capillaries by escaping through the stomata, which Becklinhauses has shown to exist between the endothelial elements.

*The Throat.* If in the nasal passages the edema is so great that the infant cannot breathe through the nose, respiration must of necessity be carried on through the mouth. This will soon cause dryness of the fauces. This condition invites extension of the edema to these parts, as well as occasions ear-trouble, for the dryness will induce frequent acts of deglutition. The action of the soft palate during deglutition, the nasal passages being full of muco-purulent secretion, will force some of the secretion up to the Eustachian tubes, to the positive injury of the middle ears and their contents. The edema of the throat is shown by the narrowing of the fauces as well as by partial enlargement of the tonsils.

*Mouth-Breathing.* This is the age of the commencement of what is called "the mouth-breathing habit." It is seen from what has been said that this symptom is a *consequence*, and not a cause, of nasal inflammation. Here the relative positions of cause and effect are exceedingly important. Give the patient normal breathing room through both nasal cavities, and mouth-breathing will be discontinued in a short time. It is an unnatural process, and always acquired from necessity. Until this is done, it is foolish to say "shut your mouth and save your life," unless the user of this term means to say, "free your nasal passage of chronic inflammation, its growth and other sequences," so that you can "close your mouth and save your life; that would most certainly be consistent with facts. If the nasal inflammation is so severe as to interfere with the nutrition of the bones of the face, the child will acquire a peculiar physiognomy, due to the arrest of the development of these bones, the open-mouth breathing aggravating the peculiarity. I have works on my shelves that say this peculiar facial expression is due to mouth-breathing, which is a very serious mistake, as it obscures the indications for its proper, and it may be its successful, treatment.

*Aphasia.* Fortunately it is quite rare that nasal inflammation is so excessively severe as to extend into the brain so as to cause a disability of speech. The disability may be only a slowness in learning to speak, or slovenly way of pronouncing words, or the disability may be so great that there may be a total inability to pronounce one word that could be understood by any one save the mother. The subject may never learn to pronounce one word; making himself intelligible only by peculiar inflections of high or low tones, and by laughing and crying. With this disability there is nearly always an atrophy of the bones of the face and mouth-breathing. I have had, since 1871, seven such cases, from seven to twelve years of age—that is, with total inability to speak one intelligible word. It was not the least difficult in each of the cases to trace back to the time when the nasal inflammation, during infancy, was doing its mischief; the nasal disease in all of these cases was distinctly remembered by the parents.

That the nasal inflammation extended, in these cases, to the third convolution of the brain and the island of Reil—the center of speech—was evident upon close and careful investigation of each case. The auditory nerves were somewhat affected in all the cases, but hearing was not very dull, for all the patients could hear the usual voice at the distance of from two to four feet when their backs were turned to the speakers, and five of them gave abundant evidence of their ability to understand what was said to them. There was evidence of defective hearing in infancy, in the marked projection of the pinna of the ears. The sight on casual examination was perfect. The facial nerves were unaffected as far as could be ascertained by common tests. The sensory nerves were nearly normal, but all of the patients had locomotor ataxy to a greater or less degree. Some could not walk at all with a handkerchief over their eyes. Others staggered so that they fell when pushed into a walk. Not one of them would willingly take a single step with a handkerchief thrown over the head so as to cover the eyes. The absence of the ability to speak under these circumstances clearly points to the brain as the organ injuriously affected, and as each one has been in *perfect health* since their recovery from the severe illness that affected their nasal passages, it shows that the nasal inflammation had extended its injurious influence to the third frontal convolution and the island of Reil.

*The Ears.* Up to the third year of age the abnormal nasal secretion has at first but little pus in it. The injury that the secretion does is by the excessive quantity that is formed, and because of its fluidity. The quantity is so great that the pharyngo-nasal cavity is sometimes completely filled with it, so that as the air goes up into the Eustachian tubes, the secretion also is drawn with it into the middle ear. This condition shortly—in a few hours at most—creates so much distress as soon to bring about serious ear-symptoms, causing acute tympanitis and myringitis, and unless perforation of the membrane tympani takes place, serious brain symptoms will soon follow, which may last but three or four days before death takes place. In fatal cases pneumonia frequently assists in taking life, the nasal secretion being full of pneumococci.

I have made but few post mortem examinations of infant's heads, but before making these I had predicted in each case serious ear complications, for the reason that I saw purulent secretion flowing from the nostrils, and because the infants had strabismus and nystagmus before they died, and the prediction was verified. Serious disease of the brain was also discovered in each case. In not one of the infants was there the least otorrhea, nor was there any redness or swelling about the outer ear, yet a greenish pus was found in each affected middle ear. It is plainly seen that in each of these cases death was occasioned by an excessive quantity of fluid secretion in the pharyngo-nasal cavity. That this condition is not very uncommon will be inferred from the following quotations: Troeltsch says, in his work on "Diseases of the Ear in Children," page 39, that an investigator examined ears of only new-born children—twenty-four in all—the oldest age twenty-five days, "and found the tympanum empty in six, while in eighteen the cavity contained fluid, and in four of the eighteen the fluid was true pus." Troeltsch himself, without knowing this, examined "forty-seven petrous bones taken from twenty-four *unselected* children." Of the forty-seven ears he found only *eighteen that were normal;* the other *twenty-nine* showed various degrees of diseased action, rarely of a mucous character, mostly masses of purulent matter. "The drum membrane was never perforated, . . venous hyperæmia and congestion of the brain were always present when that part was examined." Another investigator found in *eighty* ears only *fourteen that*

*were normal,* the other *sixty-six* abnormal. Another investigator: "From about *two hundred and thirty* accurately described cases, the tympanic cavity was normal in only thirty." I may be mistaken, but I think that every one of these children's ears was affected through the Eustachian tube, and that from disease of the pharyngo-nasal cavity, and this again from nasal disease occasioned by colds.

The frequency of the coincidence of sudden attack of serious ear-disease and pneumonia suggests a relation of the one to the other, and I believe that the fact that nasal secretion of a person who has a severe cold in the head has pneumococci in it shows this relation and demonstrates the origin of the pneumonia. Of course the cold and the ear-disease prepared the system so that inflammation of the lungs more readily took place. I know a physician who said he always gave rabbits pneumonia when he gave them a hopodermic injection with his saliva. I remarked to him: "You may have had a cold in your head at the time; if so, your saliva, more than likely, had pneumococci in it, as every slide with this kind of saliva in it that I have examined since 1888 had this kind of bacteria in it." The nasal secretion, during a cold in the head, is frequently drawn down the pharyngo-nasal cavity, and then into the mouth and spat out. At these times the saliva is sure to be impregnated with the various kinds of bacteria that are found in the nasal cavities. Every injection of this kind of saliva will give a rabbit pneumonia; but, according to my experience, injections of saliva of persons who have *no* colds in the head will *not* cause a pneumonia in the rabbit. I admit that my experience in this respect is limited. I have made but about twenty hypodermic injections of saliva of healthy persons, and most of the injections were made into four rabbits. In one rabbit I made seven injections of saliva of seven different healthy persons. After this I made an injectian of saliva from a person who was suffering from a severe cold, and killed the rabbit, it dying of pneumonia. To speak with positiveness on this matter one should make a large number of carefully noted injections of both kinds of saliva.

*Deaf-Mutism.* This is the age of the little sufferer when the disease of the ears will transform him into a *deaf-mute.* The inflammation of the middle ear, originating from the irritating effect of the secretion that passed up the Eustachian tubes, has been severe enough to cause the escape of serum that is fibrinous; this has filled the tympanum as well as caused ankylosis of the ossicula auditus. The inflammation must also have been severe enough to have extended into the external ear and so injured the auditory nerve as to render it useless.

It is evident to every medical man that severe inflammation, such as above briefly described, *must* produce other deranging effects in the infant's organism. Some symptoms that are purely secondary to nasal disease, are named as symptoms of another disease by physicians who have given but little attention to mucositic inflammation of the nasal passages. One of the most common things is to see an infant that has been suffering for some weeks from nasal inflammation, treated for malarial fever, and as the remedies that are beneficial in malarial fever are also relieving to an advanced nasal inflammation, the recovery is taken as proof of the correctness of the diagnosis. But a close examination of the most prominent symptoms of the two diseases will show that there is a marked difference.

In cases with symptoms resembling those of malarial fever, but due to nasal inflammation, there is not engorgement of the spleen nor enlargement of the liver, as is found in about ninety per cent. of malarial cases. In nasal cases the brain and spinal cord show marked indications of diseased action; not so in malarial cases. In nasal cases there are no regular paroxysms due to exposure, while in malarial fever paroxysms are regular. In marial fever, during the period of incubation, there are no symptoms, the patient appearing in *perfect* health; in nasal cases, there is *no time* in which the patient is not under the debilitating influence of nasal inflammation. In nasal disease, on a bright, clear day the patient is improved in health; with the malarial case, a bright, clear day does not check his paroxysms.

If a nasal case has more dangerous symptoms than those usually seen in malarial cases, the complaint—according to what I have seen in several instances—is called typho-malarial, or typhoid, or infantile remittent fever; but here again we have distinguishing features that will indicate the true nature of the case: (a) The fact of the secretion being seen in the nasal passages, but not seen in typhoid. (b) Intestinal symptoms, other than hemorrhage, are absent in nasal cases, but not in typhoid. (c) Bronchitis is present in nasal cases, but not in typhoid. (d) Nervous symptoms are marked and constantly present in nasal cases, but not in typhoid. (e) Intestinal hemorrhage is common in typhoid, but not in nasal cases. (f) In typhoid the temperature has more of a remittent type than in nasal cases. (g) In typhoid the infant's recovery may take place in one night; in a nasal case such rapid recovery does not take place.

It is very urgent that this subject should be more carefully and thoroughly investigated than it has been up to the present time. Until we are allowed to make more post mortem examinations, not of the poverty-stricken that flock to the clinics, but of the infants that die among the well-to-do classes, until this has been done for from five to ten years, children will necessarily be under the care of those who *guess* that maybe this, or maybe that, will be good for them when their nasal passages and ears are seriously affected.

