# Correction

**Published:** 1896-06

**Authors:** 


In our last issue, page 147, last line, *limitation* should have been *irritation.*

